# *Sacs* R272C missense homozygous mice develop an ataxia phenotype

**DOI:** 10.1186/s13041-019-0438-3

**Published:** 2019-03-12

**Authors:** Roxanne Larivière, Nicolas Sgarioto, Brenda Toscano Márquez, Rébecca Gaudet, Karine Choquet, R. Anne McKinney, Alanna J. Watt, Bernard Brais

**Affiliations:** 1 Department of Neurology and Neurosurgery, Montreal Neurological Institute, McGill University, Room 622, 3801, University Street, Montreal, Québec, H3A 2B4 Canada; 2 Department of Biology, McGill University, Montreal, Qc Canada; 3 Department of Human Genetics, Montreal Neurological Institute, McGill University, Montreal, Qc Canada; 4 Department of Pharmacology and Therapeutics, McGill University, Montreal, Qc Canada

**Keywords:** ARSACS, Purkinje cell, cerebellum, Sacsin, *SACS*, Ataxia, Mouse model

## Abstract

Autosomal recessive spastic ataxia of Charlevoix-Saguenay (ARSACS [MIM 270550]) is an early-onset neurodegenerative disorder caused by mutations in the *SACS* gene. Over 200 *SACS* mutations have been identified. Most mutations lead to a complete loss of a sacsin, a large 520 kD protein, although some missense mutations are associated with low levels of sacsin expression. We previously showed that *Sacs* knock-out mice demonstrate early-onset ataxic phenotype with neurofilament bundling in many neuronal populations. To determine if the preservation of some mutated sacsin protein resulted in the same cellular and behavioral alterations, we generated mice expressing an R272C missense mutation, a homozygote mutation found in some affected patients. Though *Sacs*^*R272C*^ mice express 21% of wild type brain sacsin and sacsin is found in many neurons, they display similar abnormalities to *Sacs* knock-out mice, including the development of an ataxic phenotype, reduced Purkinje cell firing rates, and somatodendritic neurofilament bundles in Purkinje cells and other neurons. Together our results support that *Sacs* missense mutation largely lead to loss of sacsin function.

**Electronic supplementary material:**

The online version of this article (10.1186/s13041-019-0438-3) contains supplementary material, which is available to authorized users.

## Introduction

Autosomal recessive spastic ataxia of Charlevoix-Saguenay (ARSACS [MIM 270550]) was first described in the French Canadian population in 1978 [1]. Since then, ARSACS cases have been reported worldwide [2] (www.lovd.nl). The original French Canadian ARSACS clinical phenotype consists of a childhood onset progressive spastic ataxia accompanied by sensory-motor polyneuropathy and retinal thickening [3, 4]. French Canadian ARSACS patients become wheelchair-bound on average by the age of 41 and life expectancy is reduced to 61 years [5]. Pathological findings of post-mortem examination of two male ARSACS patient brains show atrophy of the anterior vermis associated with Purkinje cell death, while the cerebellar hemispheres are much less affected [6–8] ARSACS is the second most common form of recessive ataxia in the Netherlands and Northern UK [9].

The *SACS* gene mutated in ARSACS is located on human chromosome 13q12 and encodes sacsin, a 4579 amino acids protein. The enormous size of the *SACS* gene and its translated protein has considerably hindered functional studies. Sacsin is a multi-domain protein containing an N-terminal ubiquitin-like domain shown to bind to the proteasome [10]. Towards the C-terminus, sacsin contains a DnaJ domain [10, 11] immediately followed by a higher eukaryotes and prokaryotes nucleotide-binding C-terminal (HEPN) domain [12]. The DnaJ domain was demonstrated to bind Hsp70 and to be functional in complementation assay technique using Hsp70 chaperone [10, 11]. The HEPN domain was recognized by bioinformatics analysis to exist in a single copy in the human genome that is exclusive to sacsin [13]. The HEPN domain also mediates dimerization of sacsin [12]. In 2013, Romano et al. described using bioinformatics, three large internal homologous repeating regions, which they named SIRPT1, 2 and 3 [14]. Each SIRPT is divided into sub-repeats namely sr1, sr2, sr3 and srX. The second repeat lacks srX, making SIRPT2 smaller than the others. Each sr1 contains a well-recognizable HATPase_c (Histidine kinase-like ATPases) domain homologous to the nucleotide-binding domain (NBD) of the Hsp90 chaperone. The region combining the sr1 and sr2 corresponds to Anderson and colleagues’ SRR supradomain [11], which possesses ATPase activity. A missense pathogenic mutation, D168Y, within the sr1 completely abrogates the ability of this domain to hydrolyse ATP. The sr1 sequence in all three SIRPT domains are sites for a number of pathogenic homozygote missense mutations, or single mutations combined on the other allele with macrodeletion, frameshift and stop mutations: D168Y, T201K, R272C, R272H, R276C, L308F, P1583R, H1587R, R1645Q and R2703C [9, 14–19]. Using crystal structure analysis of the SIRPT1-sr1 encoding construct, the missense mutations R272C, R272H and T201K were demonstrated to affect the structure of protein folding and/or stability of the peptide, whereas the D168Y mutation likely affects chaperone activity by interfering with ATP binding [20]. These findings suggest that distinct mutations will variably affect sacsin protein function.

The organization of the sr1 and sr2 domains matches the structure of an Hsp90-like protein [11, 14, 21], where the sr1 represents the ATP binding domain, whereas the sr2 acts as the Hsp90-like putative middle domain containing an arginine residue accepting phosphate after ATP hydrolysis. As previously observed, there is indeed a phosphor-acceptor arginine in each sr2 domain of sacsin [11]. Significantly, a mutation on one such conserved arginine, namely R474C, was associated with one of the highest clinical severity score after mutations occurring in the DnaJ and HEPN domains [14]. Since, the SIRPT repeats make up more than 80% of the total sacsin protein, exploring their roles will help better understand the function of this large protein.

With this in mind, we generated a mouse model of ARSACS harbouring the missense mutation c.816C > T (p. R272C), the *SACS*^R272C^ mutation was described in homozygote state in two cases in Canada [2, 17]. Preservation of sacsin was confirmed by Western blot in lymphoblasts of one ARSACS case [2]. The null mice display an early abnormal gait with progressive motor, cerebellar, and peripheral nerve dysfunctions reminiscent of ARSACS pathology. The clinical phenotype is accompanied by an early onset progressive loss of cerebellar Purkinje cells particularly in the anterior cerebellar lobules, which is later followed by spinal motor neuron loss, peripheral neuropathy and muscle atrophy. Loss of sacsin results in distinctive neurofilament (NEF) accumulations most notably in Purkinje cells, deep cerebellar neurons, layer 5 pyramidal cells, thalamic and pontine neurons. Here, we show that R272C homozygous animals develop a clinical and pathological phenotype comparable to the one observed in *Sacs*^−/−^ animals despite preservation of some sacsin expression.

## Results

### Gene targeting

**Fig. 1:**
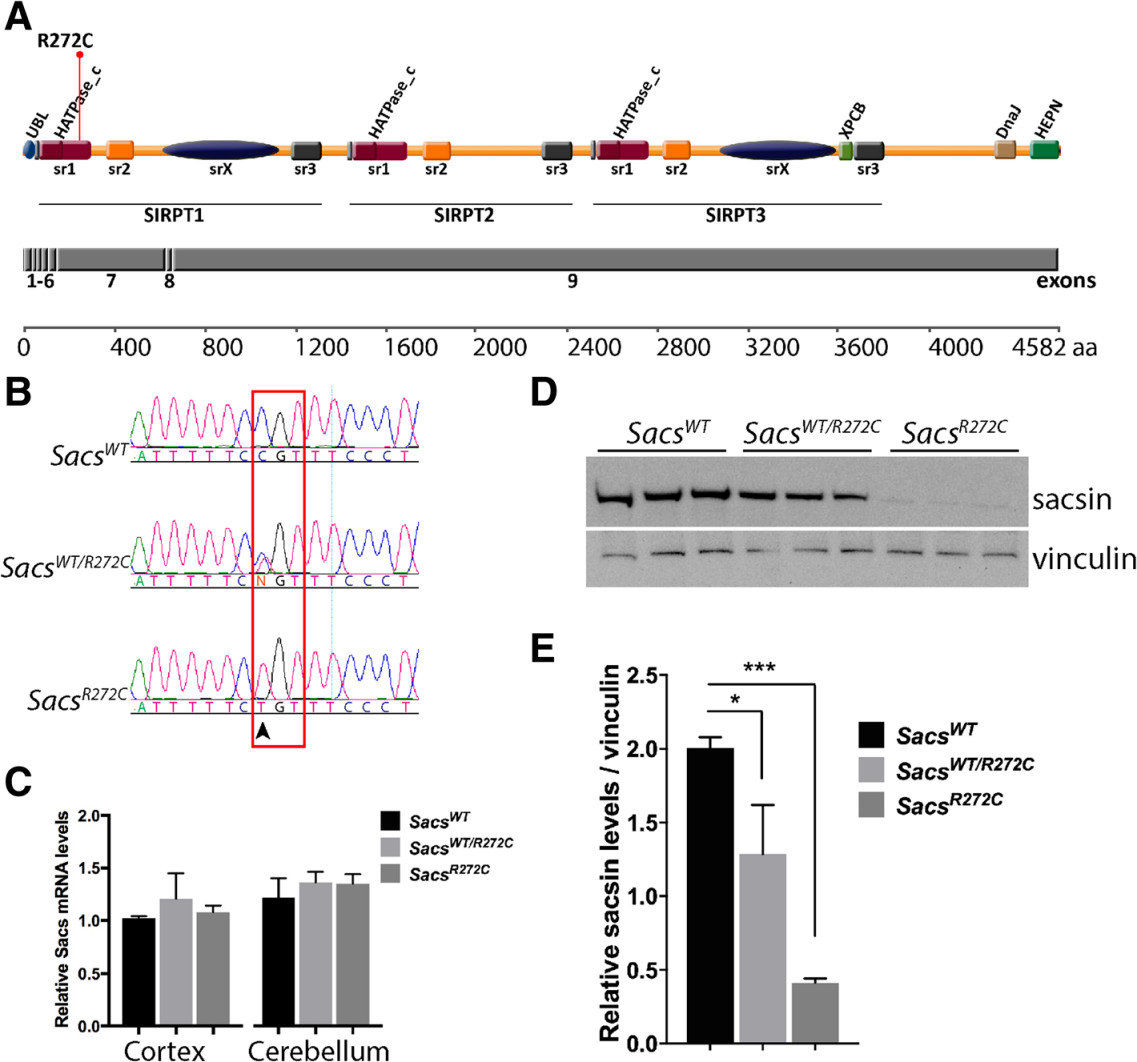
R272C *Sacs* knock-in mice. (**a**) Schematic representation of mouse sacsin protein domains as suggested by Romano et al. (2013), and exons as revealed by gene browser Ensembl. The R272C mutation lies at the end of the first sr1 domain and is localized in exon 7. (**b**) Sanger sequencing of mouse tail genomic DNA extracted from *Sacs*^R272C^, heterozygous (*Sacs*^WT/R272C^) and control (*Sacs*^WT^) mice. The C to T mutation changes the amino acid from arginine to cysteine. (**c**) qRT-PCR of RNA purified from cortex and cerebella showing no significant difference between *Sacs* RNA levels of R272C, heterozygous and control mice. (**d, e**) Western blot analysis reveals significant reduction in mutant sacsin protein levels in cerebella of *Sacs*^R272C^ and heterozygous mice (**d**). Protein level quantification demonstrates a significant decrease in mutant sacsin protein in *Sacs*^R272C^ (79%) mice and *Sacs*^WT/R272C^ (35%) compared to control (**e**). Data represent means ± SEM, *n* = 3 mice per group; *P < 0.05, **P < 0.01, ***P < 0.001 (unpaired *t*-test)

The choice of the R272C missense mutation was based on reports of human homozygous cases in Canada and the actual presence of residual amount of mutated protein in patient lymphoblasts [2, 17]. Our hypothesis is that residual amount of mutated sacsin could retain some sacsin function and influence the severity of the phenotype. A novel knock-in (KI) mouse model harbouring the c.816C > T (p.R272C) mutation in mouse *Sacs* exon 7 was generated by Ozgene using traditional gene targeting techniques (Additional file 1: Figure S1). This mutation lies at the end of the homologous HATPase_C domain in the first sr1, as described by Romano et al. (Fig. 1a) [14]. The mutation was confirmed by Sanger sequencing of mouse tail genomic DNA (Fig. 1b), demonstrating the C to T mutation at position c.816 in heterozygous and homozygous animals. *Sacs* mRNA levels were quantified by qRT-PCR using RNA extracted from cortex and cerebellum (Fig. 1c). *Sacs* mRNA levels were comparable to controls in heterozygous and homozygous *Sacs*^R272C^ brain tissues (Fig. 1c). However, mutated sacsin protein levels were significantly reduced compared to controls. Homozygous animals had only a residual amount of 21% of mutated sacsin whereas heterozygous animals had 65% compared to normal sacsin protein levels (Fig. 1d,e). These results strongly suggest that R272C mutated sacsin is unstable and more rapidly degraded.

### Ataxia, motor deficit and muscle weakness of *Sacs*^R272C^ mice

**Fig. 2:**
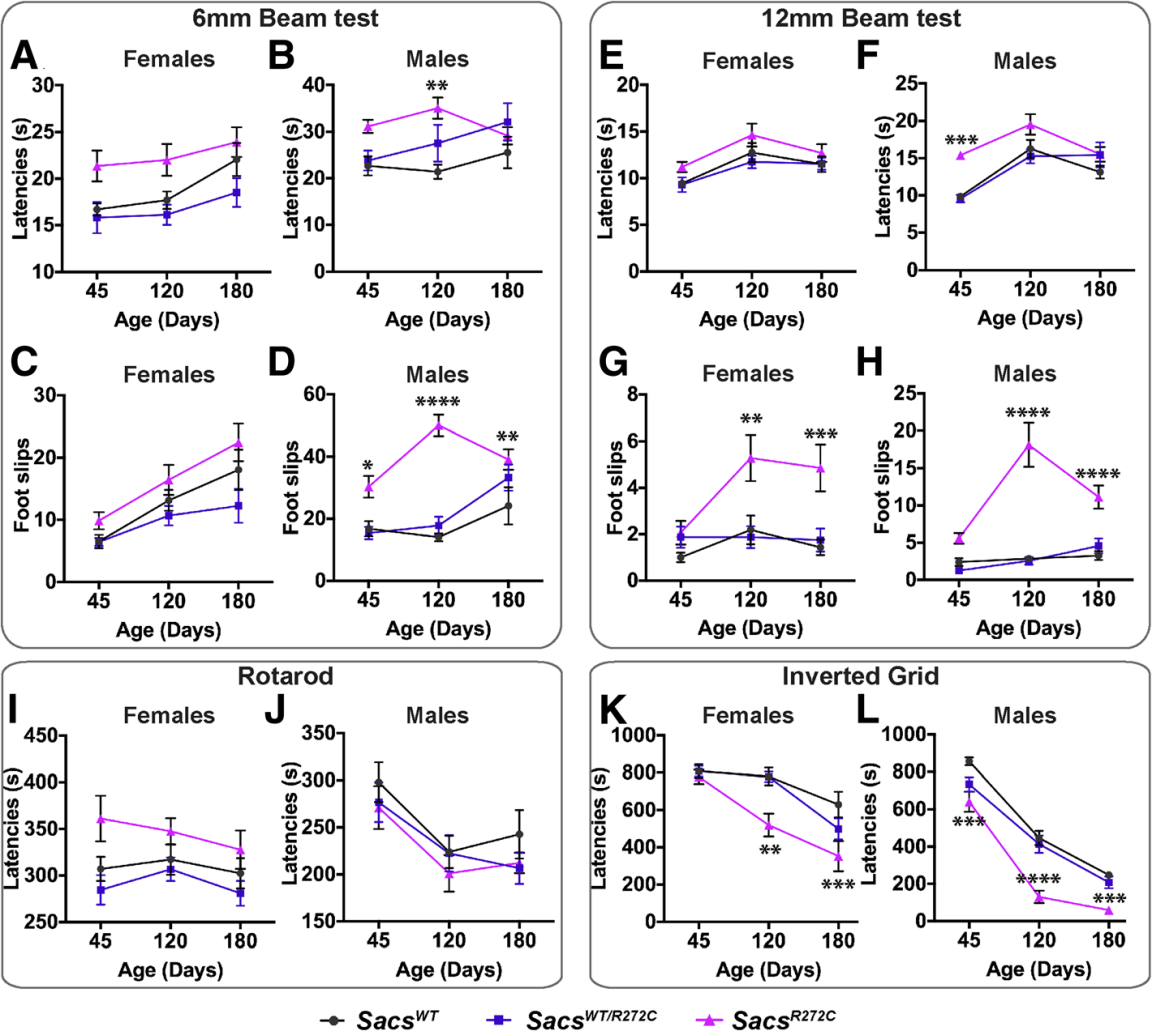
*Sacs*^R272C^ mice display balance deficit and muscle weakness. (**a–h**) Results of balance beam tests of motor coordination for a cohort of mice tested at 40, 90 and 180 days of age. Significant deficits in *Sacs*^R272C^ male mice on the 6 mm beam test depicted by increased latencies in crossing the beam (**b**) and increased number of foot slips (**d**). Significant balance deficit for females *Sacs*^R272C^ is mostly observed by increased number of foot slips on the 12 mm beam (**g**). No significant difference in performance is observed on the accelerating rotarod between groups (**i, j**). Inverted grid test of mice at 50–365 days (**k, l**). Significant muscle weakness is observed in *Sacs*^R272C^ females starting at 120 days of age compared with control mice, whereas *Sacs*^R272C^ males show significant muscle weakness starting at 45 days. Data are presented as means ± SEM of three independent trials (*n* ≥ 14 females and *n* ≥ 15 males per group). R272C versus WT: *P < 0.05, **P < 0.01, ***P < 0.005; (two-way ANOVA with repeated-measures followed by Tukey’s post hoc comparison)

Similar to the *Sacs*^*−/−*^ mice, homozygous *Sacs*^R272C^ mice are born in a Mendelian ratio, breed normally and have a normal lifespan, with many mice surviving more than 2 years. We quantified motor performance of the animals on a series of tests measuring balance, motor coordination and muscle strength. A significant difference is observed between *Sacs*^R272C^ and control animals on the balance beam as early as 45 days of age. This is mostly evident in males, but also observed in females at later time-points starting at 120 days of age, where the *Sacs*^R272C^ animals increase their number of foot slips when performing the task (Fig. 2 a-h). No significant variation is seen in the amount of time to perform the balance beam test for the *Sacs*^R272C^ females compared to controls. However, *Sacs*^R272C^ males do show significant difference in performance, though they tend to perform better at the 180 day time point (Fig. 2b, f). The differences in balance beam performance observed between males and females are most likely due to animal size and differences in muscle strength independently of genotype, which is solicited during this motor coordination test. Performance on the rotarod test, used to assess more general motor coordination, demonstrates no significant difference between the groups (Fig. 2 i, h). When assessing muscle strength using the inverted grid test, we detect significant and progressive muscle weakness in both female and male *Sacs*^R272C^ mice compared to age-matched controls starting at 120 days of age (Fig. 2. k, l). Altogether, these results demonstrate that *Sacs*^R272C^ mice display an early balance deficit and muscle weakness comparable though possibly a little milder to the one observed in the *Sacs*^−/−^ mice.

### Purkinje cell loss in *Sacs*^R272C^ mice

**Fig. 3:**
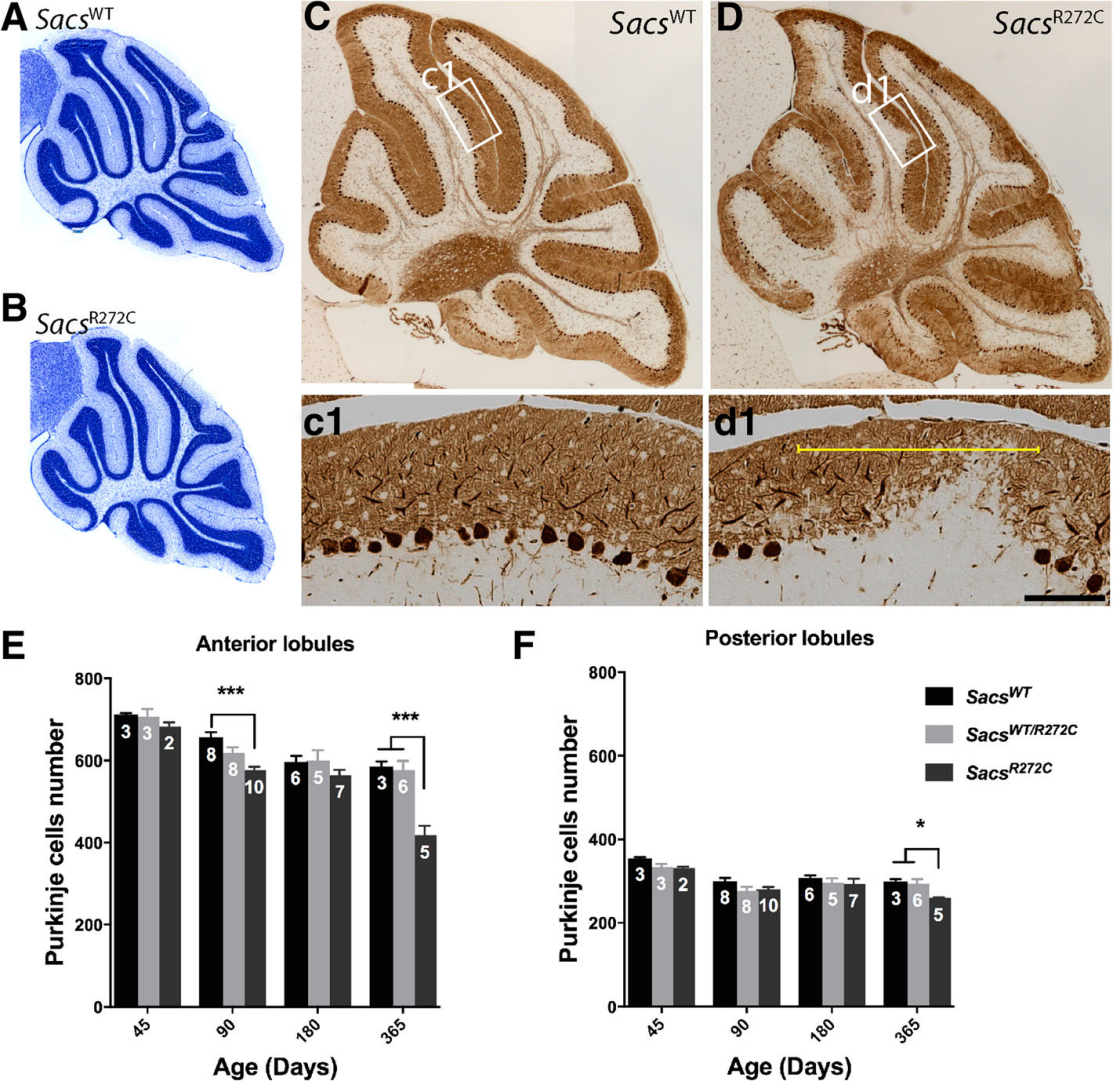
Progressive Purkinje cell loss in *Sacs*^R272C^ mice. (**a, b**) Nissl stain of 45 day-old vermal cerebellar sections show normal cerebellar structure and lobulation in *Sacs*^R272C^ mice (**b**) compared to control (**a**). (**c-d**) Calbindin immunolabeling on vermal sagittal brain sections from 300 day-old mice. *Sacs*^R272C^ mice display cerebellar PC loss **(d1,** yellow bracket) compared to age-matched controls (**c1**). (**e** and **f**) Neuronal cell counts in the anterior (I to VI) and posterior (VII to X) lobules at different ages, demonstrate significant loss of Purkinje cells in *Sacs*^R272C^ mice starting at 90 days of age in the anterior lobules (**e**). Some neuronal cell loss is observed in the posterior lobules at 365 days (**f**). Data represent means ± SEM, number of mice per group is indicated in each bars. ***P < 0.001, *P < 0.05 (two-way ANOVA with repeated-measures followed by Tukey’s post hoc comparison). Scale bar in d1 = 100 μm

Our previous studies of *Sacs*^−/−^ mice demonstrated a progressive Purkinje cell loss in cerebellar anterior lobules with little loss of Purkinje cell in the posterior lobules, mimicking the differential regional neurodegeneration observed in ARSACS patients [22]. In order to quantify Purkinje cell loss progression in our new mouse models, we stained sagittal cerebellar sections from animals at different time points (45, 90, 180 and 365 days) using Nissl stain to visualize neuronal nuclei. As it was observed for *Sacs*^−/−^ mice [22], Nissl-stained sections from 45 day-old *Sacs*^R272C^ demonstrate normal cerebellar structure and lobulation compared to age-matched controls (Fig. 3a,b). *Sacs*^R272C^ mice do, however, present significant Purkinje cell loss in the anterior lobules starting at 90 days of age and progresses through one year of age (Fig. 3e). Cerebellar sections from one-year-old *Sacs*^R272C^ mice, immunolabeled with calbindin antibody demonstrate regions of neuronal loss in the Purkinje cell layer (Fig. 3D and d1 yellow bracket). We also observed a significant decrease in Purkinje cell numbers in the posterior lobules in one-year-old *Sacs*^R272C^ mice (Fig. 3f). This result might reflect a deleterious effect of a decreased sacsin function in distinct posterior cerebellar PC.

### *Sacs*^R272C^ mice exhibit reduction Purkinje cell firing frequency

**Fig. 4:**
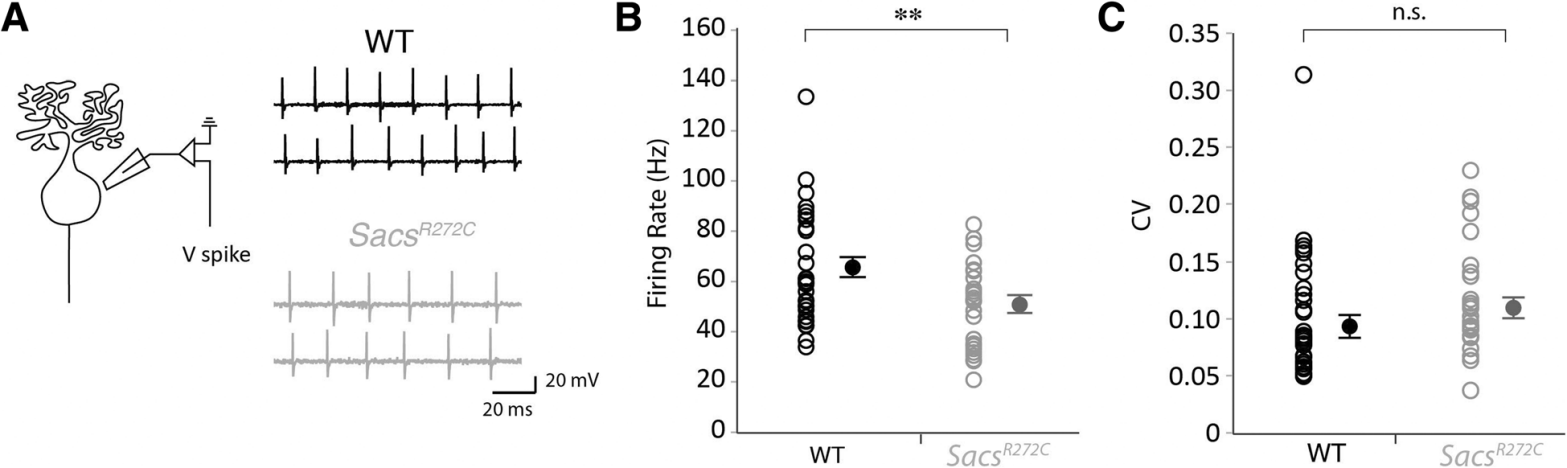
Reduced Purkinje cell firing frequency in mice with R272C mutant sacsin. (**a**) Schematic representation of Purkinje cell loose-cell attached recording configuration (left) and sample traces for WT (top right, black) and *Sacs*^R272C^ mice (bottom right, grey) Purkinje cell action potential recordings. (**b**) Purkinje cell firing rate is significantly reduced in *Sacs*^R272C^ mice, while (**c**) the precision of firing, as reflected by CV, is unaffected. n.s. = *P* > 0.05; ** = *P* < 0.01

Next we wanted to determine whether alterations in Purkinje cell spiking output was observed in our *Sacs*^R272C^ knock-in mouse model as it has in the knockout ARSACS model previously characterized [23], as well as several other forms of ataxia [24–33]. We performed cell-adjacent loose-cell attached recordings from visually-identified Purkinje cells to monitor their firing properties without disturbing their intracellular milieu (Fig. 4a, left), and recorded spontaneous action potentials in 90 day-old controls and *Sacs*^R272C^ mice (Fig. 4a, right). We observed a significant reduction in firing frequency in *Sacs*^R272C^ mice compared to control WT mice (WT: frequency = 65.5 Hz ± 3.6 Hz, *N* = 4, *n* = 31; *Sacs*^R272C^: frequency = 50.4 Hz ± 3.8 Hz, N = 4, *n* = 28; significantly different, *P* = 0.0065; Fig. 4b). This ~ 25% reduction in P90 *Sacs*^R272C^ mice is similar to the reduction found at earlier ages in *Sacs*^*−/−*^ mice, where a 15% reduction was observed at P20, and a 45% reduction at P40. Decreases in spike frequency has been observed in several forms of ataxia [24–33], which in some cases has also been accompanied by a reduction in spike regularity [31]. To determine if changes in firing precision were observed in our knock-in mouse model of ARSACS, we measured the coefficient of variation (CV) of Purkinje cell action potential intervals, since a reduction in firing regularity is associated with an increase in CV [31]. We found no significant changes in firing precision in *Sacs*^R272C^ mice (WT: CV = 0.11 ± 0.009; *Sacs*^R272C^: CV = 0.10 ± 0.009; not significantly different, *P* = 0.23; Fig. 4c), consistent with our previous findings in *Sacs*^−/−^ mice [23].

Since differences in the onset of motor abnormalities have been observed in male and female mice in the past, we wondered whether the changes in firing we observe reflect differences in male and female mice. To examine this, we compared our findings in male and female mice and found that decreases in Purkinje cell firing frequency are observed in both sexes (male WT frequency = 49.0 Hz ± 2.5 Hz, *n* = 9; male *Sacs*^R272C^ frequency = 29.6 Hz ± 2.6 Hz, *n* = 8; significantly different, *P* < 0.0001; female WT frequency = 72.3 Hz ± 4.2 Hz, *n* = 22; female *Sacs*^R272C^ frequency = 56.0 Hz ± 4.2 Hz, n = 22; significantly different, *P* = 0.009, data not shown). Thus, we observe a reduction in Purkinje cell firing frequency without any change in firing precision in our *Sacs*^R272C^ mouse model, with low expression levels of mutated sacsin, that are broadly similar to changes previously reported in *Sacs*^−/−^ mice [23].

### Neurofilament (NF) accumulations in somatodendritic compartment in *Sacs*^R272C^ Purkinje cells

**Fig. 5:**
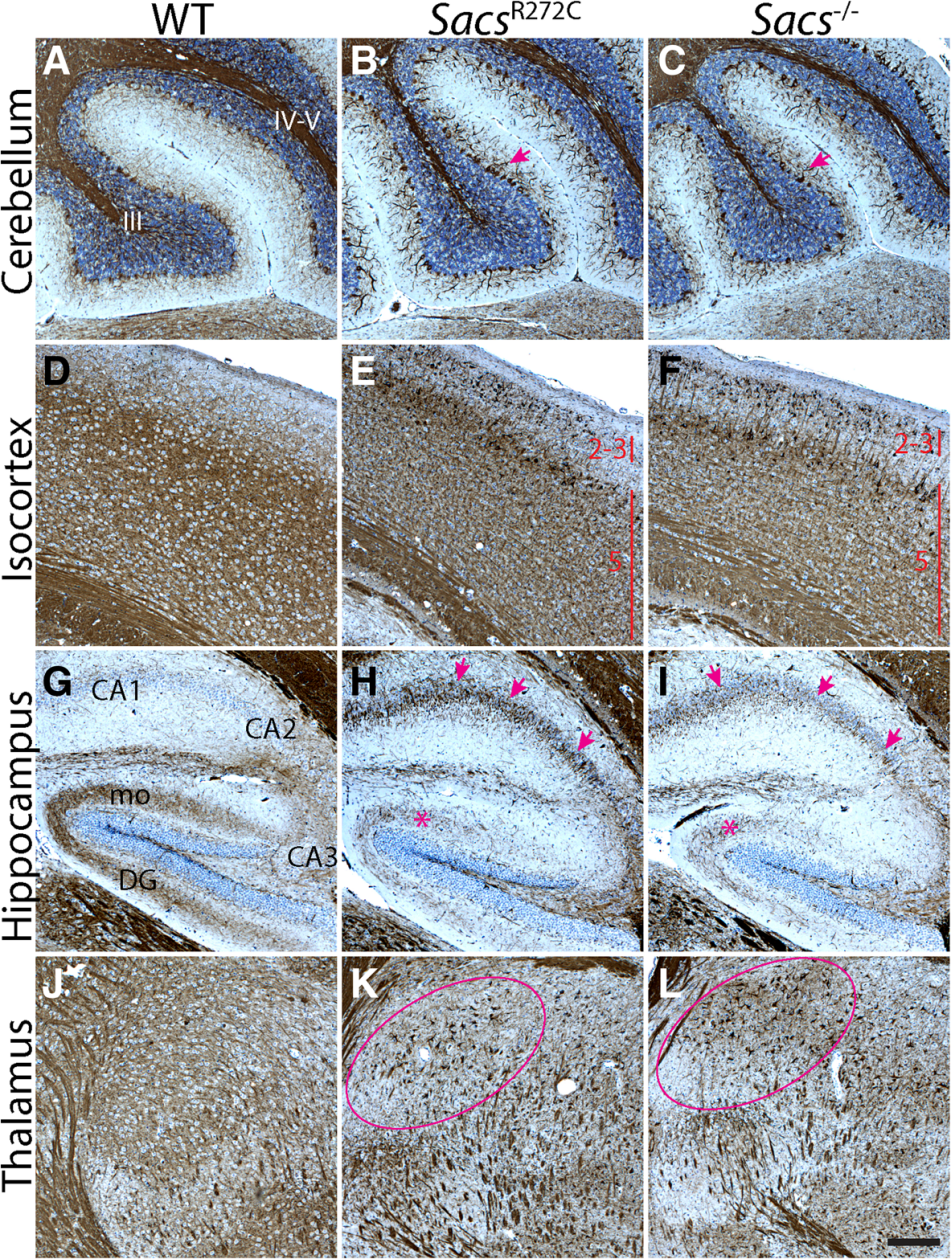
Presence of somatodendritic NFH bundles in brains of *Sacs*^R272C^ and *Sacs*^−/−^ mice. **(a-l)** Immunohistochemistry using pan-NFH antibody on sagittal brain sections from 300 day-old mice demonstrate a strong NFH labeling in the cell bodies and dendrites of several CNS neuronal populations. Among others, accumulations of NFH protein in the somatodendritic area are seen in *Sacs*^R272C^ and *Sacs*^−/−^ cerebellar PC (arrow in **b** and **c**), layer 2–3 and 5 cortical neurons (**e, f**) CA1, CA2 and CA3 hippocampal pyramidal neurons (arrows in **h** and **i**) and neurons in the thalamus (surrounded area in **k** and **l**). A decrease in NFH immunolabeling in the dentate gyrus molecular layer (mo) representing the axonal projections from the entorhinal cortex is observed in the *Sacs*^R272C^ and *Sacs*^−/−^ mice (asterisk in **h** and **i**) compared to control animals (**g**). Scale bar in L = 200 μm

**Fig. 6:**
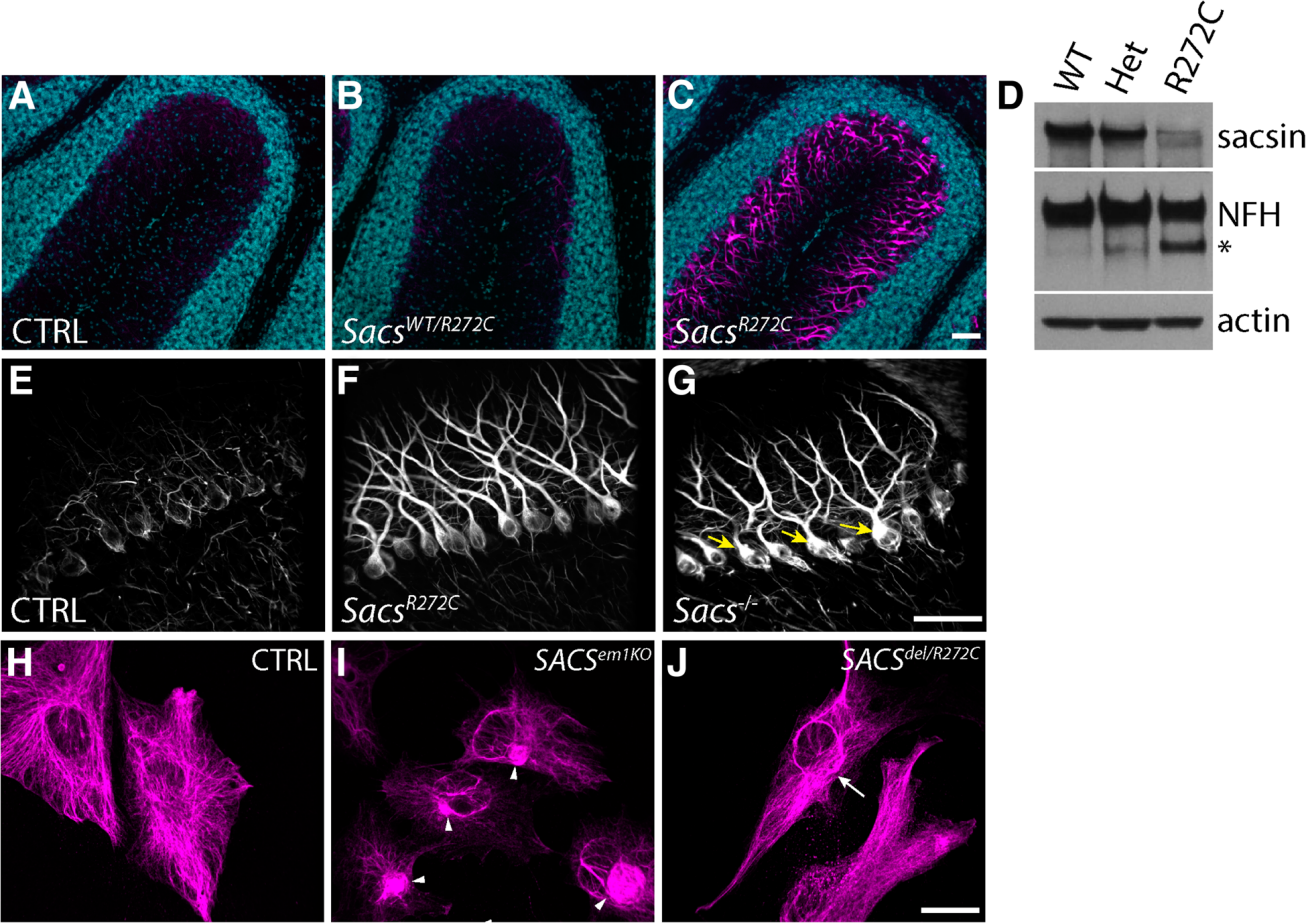
*Sacs*^R272C^ exhibit NFH bundles in Purkinje cell soma and dendrites. (**a–c**) Immunolabelings using pan-NFH antibody (pseudo-coloured in magenta) show intense NFH labeling of Purkinje cell soma and dendrites in *Sacs*^R272C^ mice (**c**) compared to control (**a**) and heterozygous (**b**) animals. Sections are counterstained with Hoechst, pseudo-coloured in cyan blue). (**d**) Immunoblots of cytoskeletal fractions extracted from cerebella of *Sacs*^WT^, heterozygous and R272C mice show increased levels of npNFH (asterisk) in *Sacs*^R272C^ homogenates when probed with pan-NFH antibody. (**e-g**) NFH bundling in *Sacs*^R272C^ is less severe than that observed in *Sacs*^−/−^ Purkinje cells. Immunolabeling for NFH reveals intense accumulation of NFH protein in *Sacs*^*−/−*^ Purkinje cell soma (**g**, arrows) compared to *Sacs*^R272C^ (**f**). No such accumulation is seen in control animals (**e**). **(h-j**) Vimentin filament bundling in ARSACS fibroblasts. Vimentin immunolabeling demonstrate intense perinuclear ball-like filament bundles in *SACS*^em1KO^ fibroblast (arrowheads, I). Vimentin filament perinuclear bundles are also observed to a lesser extent in *SACS*^del/R272C^ fibroblasts (arrows, J). Control fibroblasts demonstrate a nicely organized vimentin filament network (H). (Scale bar in C and G = 100 μm, scale bar in J = 25 μm)

Intermediate filament protein accumulations are a striking feature observed in numerous neuronal populations in *Sacs*^−/−^ and ARSACS autopsied brain, ARSACS human-derived dermal fibroblasts as well as genetically engineered knock-out cell lines [22, 34] To explore if *Sacs*^R272C^ animals present the same characteristic IF bundling as KO animals, we performed immunolabeling using a pan-neurofilament heavy (NFH) antibody on sagittal brain sections from 300 day-old mice (Fig. 5). As expected, *Sacs*^R272C^ and *Sacs*^−/−^display distinct NFH somatodendritic labeling in several CNS neuronal populations; such as cerebellar PC (Fig. 5b, c), neurons in layer II-III and V of the isocortex (Fig. 5e, f), CA1, CA2 and CA3 pyramidal neurons of the hippocampal formation (Fig. 5h, i) and neurons in the thalamus (Fig. 5k, l). NFH immunofluorescence show strong labeling in Purkinje cell dendrites as well as in cell bodies compared to very light immunolabeling in controls and heterozygous animals (Fig. 6a-c). NFH labeling in *Sacs*^R272C^ mice identifies mislocalization of NFH in Purkinje cell bodies and dendrites compared to controls where no NFH is detected in these cellular compartments (Fig. 6f). Western blots analysis revealed an increase in both, NFH protein levels, as well as, most significantly, in the non-phosphorylated form of NFH, which has also been previously reported in the *Sacs*^−/−^ mice (Fig. 6d).

Rearrangement of the intermediate filament network in sacsin-deficient cells is also observed in ARSACS patient dermal fibroblasts [34]. These cells display abnormal perinuclear accumulation of vimentin filaments [35]. To verify if the R272C mutation had similar effect on the IF network, we labeled vimentin in CRISPR/Cas9 genetically engineered *SACS* knock-out fibroblasts (*SACS*^em1KO^), in which there if no sacsin expression, and our fibroblast line derived from a patient with two distinct SACS mutations (Fig. 6h-j). On one allele, this patient bears the common French-Canadian c.8844del mutation (del) and on the other, the c.816C > T (R272C) mutation. As expected, our *SACS*^em1KO^ fibroblasts demonstrate perinuclear accumulations of vimentin often forming a ball-like shape (Fig. 6j). The vimentin network in *SACS*^del/R272C^ fibroblasts is also perturbed, with bundles of IF filaments observed surrounding the nucleus (Fig. 6i). Both these phenotypes are distinctively different from the vimentin network observed in control patient fibroblasts, however the IF bundling is less important in the *SACS*^del/R272C^ fibroblasts compared to the one in the *SACS*^em1KO^ (Fig. 6h-j).

### Mutated sacsin expression in the cerebellum and isocortex of *Sacs*^R272C^ mice

**Fig. 7:**
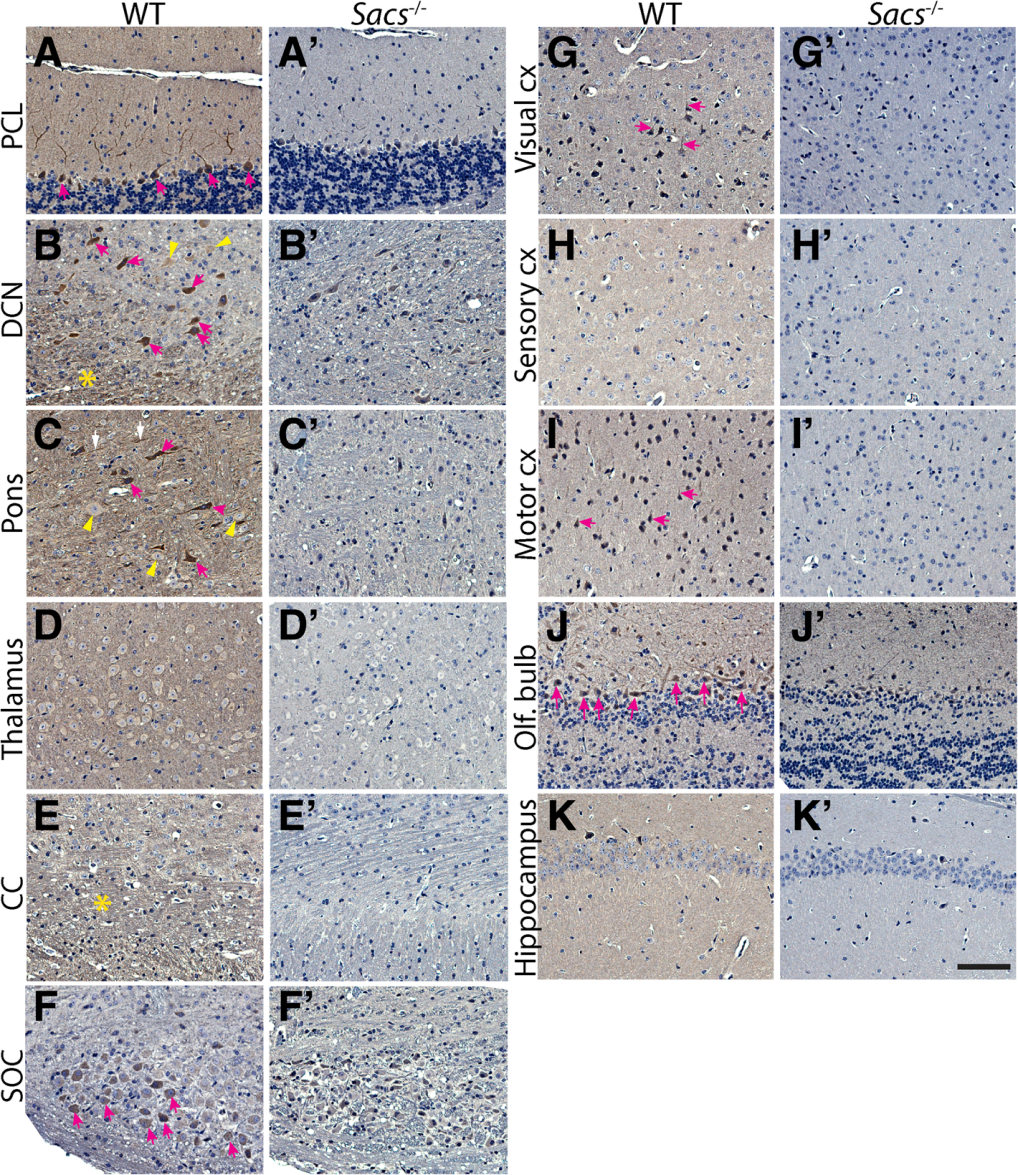
Sacsin expression in CNS neurons. **(a-k)** Immunohistochemistry using antibody against sacsin on sagittal brain sections from 300 day-old control mice demonstrate the widespread sacsin expression in several CNS populations. *Sacs*^−/−^ mice were used as negative controls (**a’-k′**). The highest expression of sacsin is seen in the cerebellum, pons and medulla. Cerebellar PC cell bodies, dendrites (**a**) and axons (asterisk in **b**) are highly labeled, a subpopulation of DCN neurons are highly labeled (pink arrows in **b**), whereas other DCN neurons are lightly labeled (yellow arrowheads in **b**). The same is observed in the pons, some isolated neurons are highly labeled (pink arrows in **c**), whereas others are lightly labeled (yellow arrowheads in **c**). In the thalamus, sacsin labeling seems restricted to the neuritis with little labeling in the neuronal cell bodies (**d**). Fiber tracts in the corpus callosum are positive for sacsin immunolabeling (**e**). Neurons in the superior olivary complex (SOC) show sacsin immunolabeling (**f**). Sacsin immunolabeling is observed in the cell bodies and dendrites of neurons in the isocortex visual (arrows in **g**) and motor areas (arrows **i**). In the somatosensory area of the isocortex, sacsin immunolabeling seems more in the neurites than in the neuronal cell bodies (**h**). Olfactory bulb mitral cells are also sacsin positive (**j**), and light labeling is observed in the hippocampal formation pyramidal cell neurites (**k**). Scale bar in K′ = 100 μm

To first determine sacsin expression in the brain, we performed immunolabeling using anti-sacsin antibody on sagittal brain sections from controls, as well as from *Sacs*^−/−^ mice serving as negative controls (Fig. 7). Immunolabeling revealed that sacsin is a neuronal protein with expression in most areas of the brain. The most extensive labeling was observed in the cell bodies, dendrites and axons of cerebellar Purkinje cells (Fig. 7a), certain neurons of the DCN (Fig. 7b) and several neurons in the pons and the medulla (Fig. 7c). Sacsin expression was also observed in cell bodies of olfactory bulb mitral cells (Fig. 7j), superior olivary complex neurons (Fig. 7f), as well as neurons in the cerebral cortex areas; visual and motor (Fig. 7g, i). In some areas, such as the thalamus (Fig. 7d), the hippocampus (Fig. 7k) and the isocortex sensory area (Fig. 7h), sacsin expression seem more restricted to neuronal processes with only very light labeling seen in the cell bodies. Sacsin immunolabeling could also be observed in fiber tracts in the cerebellum, the pons and medulla (Fig. 7c), as well as the corpus callosum (Fig. 7e). In summary, sacsin is widely expressed in the brain with some subcellular distinction from one neuronal population to the other.

**Fig. 8:**
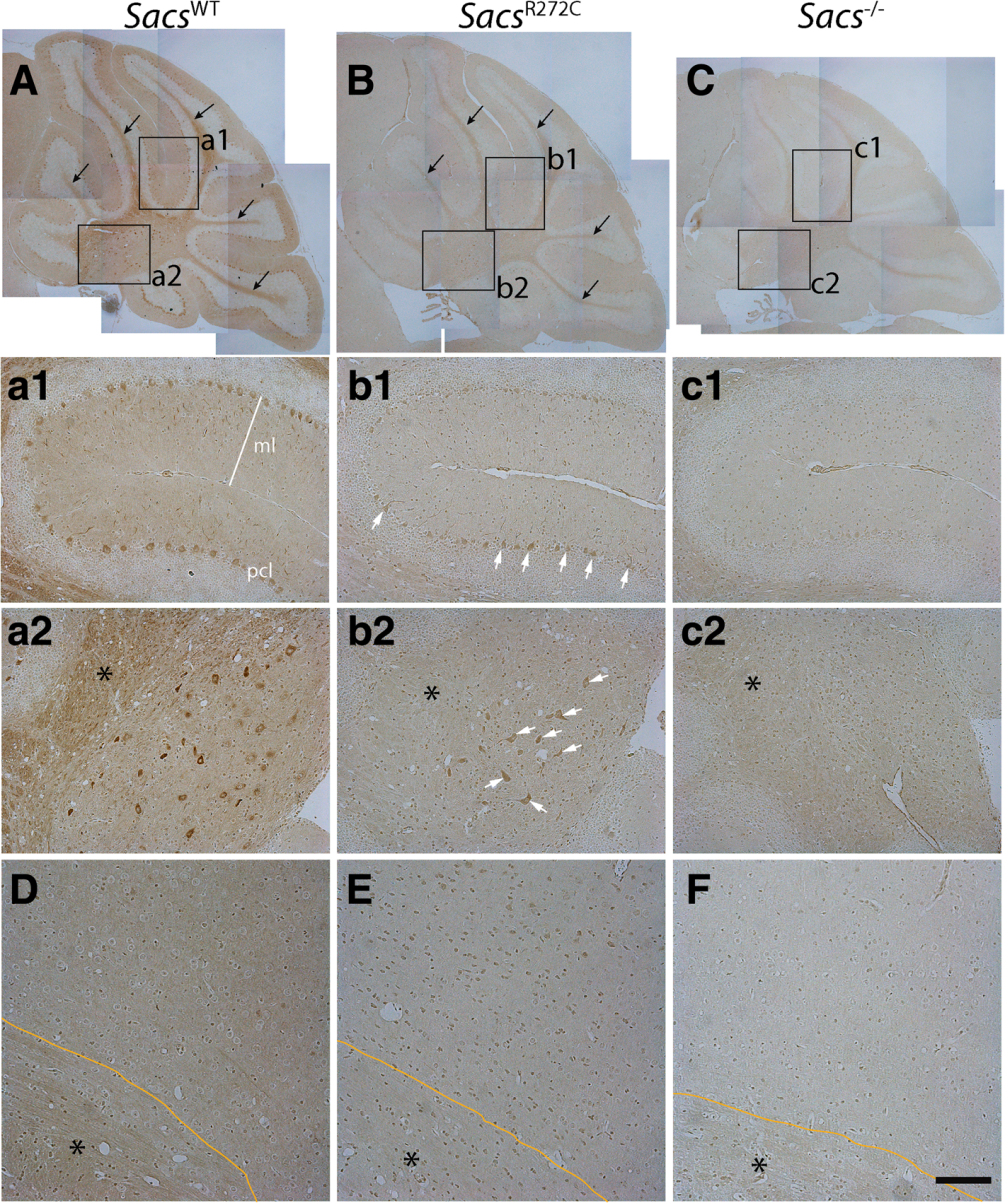
Mutant sacsin expression in *Sacs*^R272C^ mice. **(a-f)** Immunohistochemistry using antibody against sacsin on sagittal brain sections from 300 day-old *Sacs*^WT^, *Sacs*^R272C^ and *Sacs*^−/−^ mice demonstrate that some neuronal population express low levels of mutated sacsin. R272C mutant mice display an important reduction in sacsin immunolabeling in all areas of the brain, but labeling can still be observed in the cell bodies and dendrites of cerebellar PC (arrows in **b1**) and cell bodies of DCN neurons (arrows in **b2**). Scale bar in c2 = 200 μm, scale bar in F = 100 μm

**Fig. 9:**
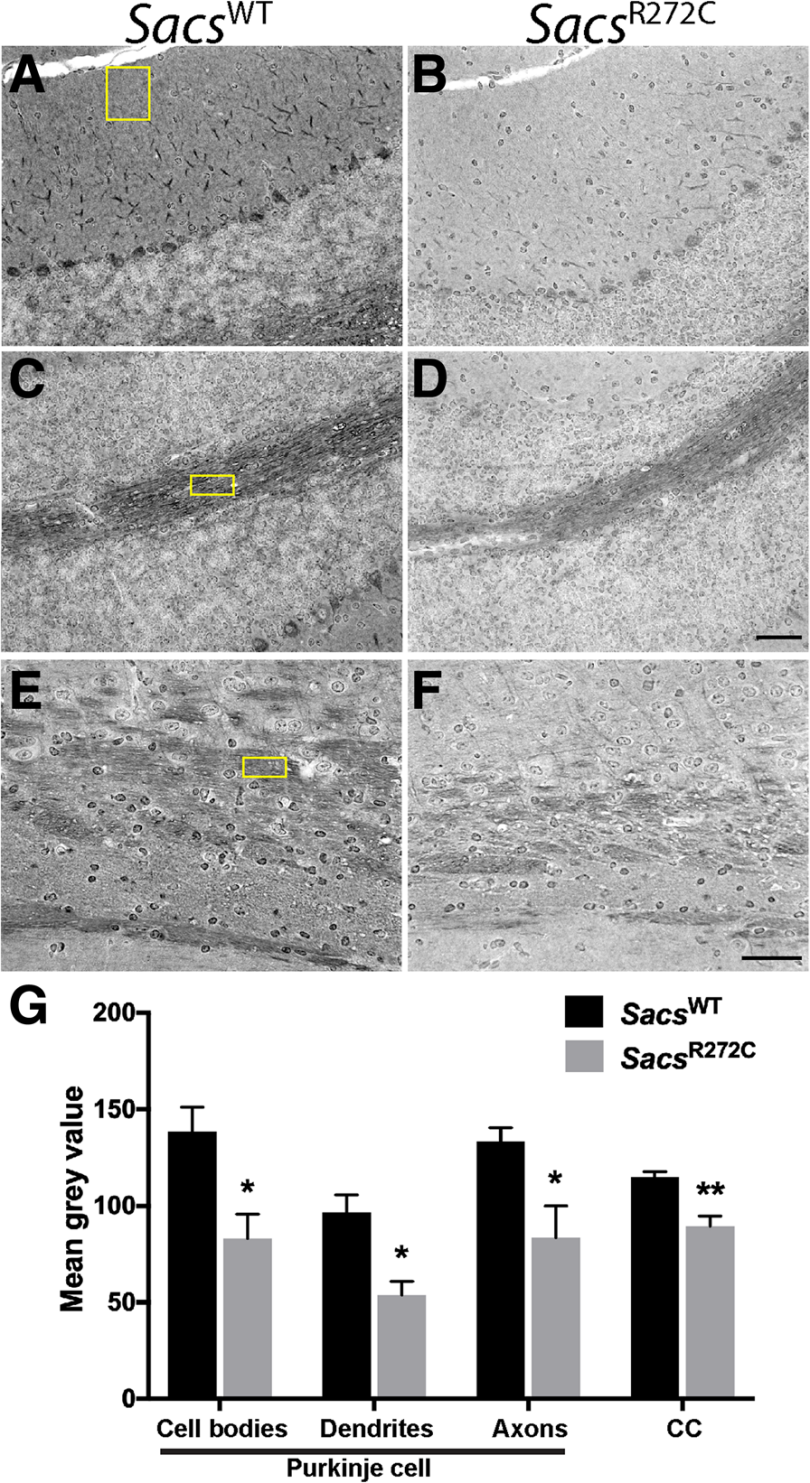
Mutant sacsin expression in *Sacs*^R272C^ mice. **(a-f)** Immunohistochemistry using antibody against sacsin on sagittal brain sections from 80 day-old *Sacs*^WT^ and *Sacs*^R272C^ mice demonstrate a reduced labeling in cell bodies (**b**), dendrites (**b**) and axons (**d**) of cerebellar Purkinje cells as well as in commissural fibers of the corpus callosum (**f**) of *Sacs*^R272C^ mice. Yellow boxes demonstrate fixed-sized regions of interest used for immunohistochemistry quantifications. (**g**) Means grey values of immunohistochemical sacsin labeling. Data represent means ± SEM, three mice per group. **P < 0.01, *P < 0.05 (One-tailed unpaired t test *Sacs*^R272C^ versus controls). Scale bar in F = 50 μm

Our *Sacs*^R272C^ mice do display an ataxic phenotype with a progressive PC cell loss, and seem to be less affected then the *Sacs*^−/−^ mice. To address whether this possible slight difference in phenotype was associated to the residual expression of mutated sacsin, we performed sacsin immunolabeling in sagittal brain sections from 300 day-old WT, *Sacs*^R272C^ and *Sacs*^−/−^ mice (Fig. 8). R272C mutant mice display an important reduction in sacsin immunolabeling in all areas of the brain, but labeling can still be observed in the cell bodies and dendrites of PC and cell bodies of DCN neurons (Fig. 8b). Indeed, sacsin immunohistochemical labeling quantification demonstrate a significant reduction of mutant sacsin immunolabeling in cell bodies, dendrites and axons of cerebellar Purkinje cells as well as in commissural fibers of the corpus callosum of *Sacs*^R272C^ mice (Fig. 9 a-f). The reduction in mutated sacsin labeling is consistent throughout Purkinje cell compartments, such as the cell body, dendrites and axons, where we observe a 40, 44 and 38% reduction respectively compared to the sacsin labeling in control mice (Fig. 9g). These results suggest that the observed phenotype is most likely attributed to an overall reduction in mutant sacsin protein levels leading to a loss of sacsin function.

## Discussion

Here we report that expression of mutated R272C sacsin protein in mice leads to a similar and potentially milder phenotype than that previously characterized in the *Sacs*^−/−^ animals [23]. However, a comparative parallel study of age-matched *Sacs*^R272C^ and *Sacs*^−/−^ mice would require too many resources for the limited insight we expect such a study would provide, considering the large spectrum of clinical severity observed in human patients, even between those carrying the same mutations. The R272C mutation was previously shown to affect proper protein fold and/or protein stability of the sr1 domain [20]. Although we did not investigate R272C mutant sacsin protein folding, we did identify a significant decrease in mutant protein levels on Western blot and immunohistochemical labelings from animal brains. *Sacs*^R272C^ mice exhibit significant balance deficit and muscle weakness detectable as early as 45 days of age. These balance difficulties preceded extensive neuronal loss, suggesting that the ataxic phenotype most likely corresponds to Purkinje cell dysfunction prior to degeneration. The progressive Purkinje cell loss was largely localized to the most anterior cerebellar lobules, just as observed in ARSACS patients. Our sacsin immunolabeling demonstrate that sacsin expression is detected in cerebellar PC across all vermal cerebellar lobules and therefore cannot account for a greater vulnerability of anterior PC in ARSACS. Other cellular or physiological properties of these cells must account for this greater vulnerability in ARSACS. We recently reported changes in synaptic input and intrinsic firing of cerebellar PC, as well as synaptic output to the DCN in *Sacs*^−/−^ mice prior to their motor coordination deficit [23]. These changes were only observed in anterior cerebellar lobules, but not in non-degenerating posterior lobules. These results support the idea that cerebellar PC across the cerebellum have distinct properties that could render certain populations more vulnerable to the absence or to deficient sacsin function. In our *Sacs*^R272C^ mouse model, we also observed Purkinje cell firing rate deficits in anterior lobules that are similar to those detected in our *Sacs*^*−/−*^ mouse model [23], supporting the hypothesis that Purkinje cell firing deficits contribute to motor coordination deficits [23, 36].

The IF cytoskeletal rearrangement observed in numerous neuronal populations in the brains of *Sacs*^R272C^ and *Sacs*^−/−^ mice, in *Sacs*^−/−^ primary neuronal cultures, in ARSACS patient-derived fibroblasts and in genetically engineered *Sacs*^−/−^ cell lines is the most striking cellular change observed to date in the ARSACS pathology [22, 34]. This phenotype appears to occur prior to motor coordination deficit and other cellular features of ARSACS, such as mitochondrial elongation and impaired transport, at least in *Sacs*^−/−^ mice [22]. We do not fully understand what causes the accumulations of non-phosphorylated NFH proteins in the somatodendritic compartment of Purkinje cells, but one explanation is that sacsin directly acts on NF assembly and/or turnover. Our recent results suggest a direct interaction of distinct sacsin domains in the regulation of IF assembly and dynamics, with certain domains, namely the SIRPT1 and DNAj domains, being capable of dismantling NF bundles in cultured *Sacs*^−/−^ neurons [37]. These results argue that sacsin could serve as an important IF protein co-chaperone.

Another possible explanation for the accumulation of NFH in the somatodendritic compartment of *Sacs*^R272C^ and *Sacs*^−/−^ neurons could be a mis-targeting or mis-sorting of proteins. Neurons are highly polarized cells exhibiting axonal and somatodendritic domains with distinct complements of cytoplasmic organelles and cytoskeletal proteins. Polarized sorting is thought to depend mainly on selective association of these cytoskeletal organelles or proteins with different microtubule motors in the pre-axonal exclusion zone (PAEZ), a specialized area within the axon hillock and the axon initial segment (AIS) [38]. Defects in this polarization could impede axonal proteins from entering the axon and stall them in the somatodendritic compartment causing neuronal defect. For example, Purkinje cell-specific knock-down of microtubule cross-linking factor 1 (*Mtcl1*) causes AIS disorganization by impairing ankyrin G localization, and loss of axonal polarity [39]. In mice, genetic disruption of *Mtcl1* results in abnormal motor coordination associated with Purkinje cell degeneration, arguing that Purkinje cells are susceptible to such deregulation of neuronal polarization [39]. Furthermore, a point mutation in the C-terminal microtubule-binding domain of MTCL1 has been found to segregate in a Japanese dominant spinocerebellar ataxia family [39]. The accumulation of NFH protein in the somatodendritic compartment of several neuronal populations in the *Sacs*^R272C^ and *Sacs*^−/−^ mice raises the possibility that sorting of dendritic and axonal proteins might be perturbed in ARSACS. Understanding the potential role of sacsin in the establishment and/or maintenance of neuronal polarity will be an important area of future study. Further studies will also be needed to elucidate whether the bundles of IF cytoskeletal proteins in ARSACS are pathophysiological and directly lead to cellular death or are simply by-products. Although it is easy to conceptualize that large bundles of cytoskeletal proteins in the neuronal cell soma and dendritic branches would physically hinder organelle and cargo protein transport in ARSAC [40], future studies are needed to confirm its pathological role.

Our results demonstrate that expression of low levels of mutant R272C sacsin in mice leads to motor coordination deficit and muscle weakness reminiscent of the human ARSACS pathology, with similar cellular deficit previously observed in the *Sacs*^−/−^ mouse model. The mutant R272C mouse demonstrate that missense *SACS* mutations are likely to interfere with sacsin function despite some low mutant protein levels, supporting that a loss of function most likely underlines its pathophysiology.

## Materials & methods

### *Sacs*^R272C^ mice generation and analysis

*Sacs*^R272C^ mice were generated by Ozgene (Bentley, Australia) on a C57BL/6J background. Targeting vector was constructed by first cloning the gene segment which includes exons 6 through 8 into PelleR B00001F7_G01 Ozgene proprietary plasmid containing a PGK-neo cassette flanked by two FRT sites, followed by site-directed mutagenesis for introduction of the R272C mutation at the beginning of exon 7. Targeting vector was completed by incorporation of 6.3Kb 5′ and 3′ homology arms. Mice were genotyped by PCR using primers: 5′- AGCAACCTGCATCATTGTAGCAGAA -3′ and 5′- GGTTTCTGGTTTGAGGCAAT -3. Total RNA from mouse cerebella and cortex was extracted with the miRNeasy kit (Qiagen) and treated with DNAse I (Qiagen) according to the manufacturer’s instructions. RNA quality was assessed on an Agilent 2100 Bioanalyzer and RNA Integrity Numbers (RIN) were routinely above 9. For qRT-PCR, 1 μg of RNA was reversed transcribed using the High Capacity cDNA Reverse Transcriptase (ThermoFisher). The following primers were used to amplify *Sacs*: 5′-CGCTGAGACCAGCTTTCC-3′ and 5′-CCAATCTTGATCCAATCAGGTATC-3′. Real-time PCR was performed in technical duplicates using FastStart Universal SYBR Green Master (ROX) (Roche) on a ViiA™ 7 Real-Time PCR System (Applied Biosystems). The ΔΔCt method was used to calculate relative *Sacs* mRNA expression, with normalization to the endogenous genes *Ppia* and *Hprt1*. *Sacs*^R272C^ mice were maintained in the C57Bl/6J background and bred and maintained under standard conditions consistent with the Canadian Council on Animal Care and approved by the University Animal Care and MNI Animal Care committees.

### Fibroblast cell lines

Control human-derived fibroblasts were obtained from the Repository for Mutant Human Cell Strains of the Montreal Children’s Hospital. *SACS*^del/R272C^ human-derived fibroblasts were obtained using the previously described protocol [41]. Briefly, patient skin punch biopsies were minced in small pieces and put in 6-well plates in complete DMEM/20% FBS (Wisent) media. Media was changed every 2–3 days. Cells were trypsinized and passaged once they reached confluence. Fibroblasts were then frozen at 1 × 10^6^ cells/ml per vial. Primary cultures were kept at low passage (p4–8). Cells were cultured in regular medium, DMEM (Wisent) with 10% FBS (Wisent) at 37 °C under 5% CO^2^ humidified atmosphere. Primary human-derived fibroblasts were immortalized at low passage as previously described [42] *SACS*^em1KO^ CRISPR/Cas9 cell line was generated following manufacturer guidelines using sacsin double nickase plasmid (sc-404,592-NIC, SCBT). Briefly, cells were nucleofected with 2μg of vectors and positive clones were selected using 1μg.ul^− 1^ puromycin (ThermoFisher Scientific). Absence of sacsin was verified by Western blotting. Genomic DNA was extracted from clones of interest and Sanger sequenced using the following primers (Fwd: CACAGTAATCATGCAAAGTCTCTATGCCTG, Rev.: ACAGAGAAACTGGTGTTTAGAGTGACTTC). Our SACS^em1KO^ Crispr/Cas9 cell line presents a 44pb duplication in exon 8 of the *SACS* gene (c.1668_1711dup) leading to insertion of a stop codon and total absence of protein (data not shown). Absence of off-target recombination was verified in silico (crispr.mit.edu). Studies using human cell lines were approved by the institutional review board of the Montreal Neurological Institute and with McGill University Research Ethics Board Committee.

### Immunolabeling

For preparation of tissue sections, mice were anesthetized with mouse anesthetic cocktail (ketamine (100 mg/ml), xylazine (20 mg/ml) and acepromazine (10 mg/ml)), perfused transcardially with 0.9% NaCl followed by 4% paraformaldehyde. Brains were dissected and post-fixed for 2 h at 4 °C in the same fixative. Tissues were then equilibrated in 30% sucrose/PBS until sectioning. Sagittal sections (35 μm) were cut using a freezing sledge microtome. Free-floating sections were processed for immunofluorescence as previously described [22]. Antibodies used were polyclonal anti-calbindin-D-28 k (Sigma, C2724), monoclonal anti-neurofilament-H (NFH) (Millipore, MAB5266), polyclonal anti-MAP2 (Abcam, ab5392).

For immunohistochemistry, mouse brains were dissected out, immersed in 4% paraformaldehyde and post-fixed for 48 h at 4 °C in the same fixative. Tissue were processed for paraffin embedding and sectioned at 4 μm in the parasagittal plan. For sacsin immunohistochemistry, sections were subjected to heat-mediated antigen retrieval in demasking solution (10 mM Tris-HCL, 1 mM EDTA, 0.05% Tween-20) at 95 °C for 35 min. Sections were allowed to cool down at room temperature for 30 min followed by inactivation of endogenous peroxidase. Sections were then incubated in blocking buffer (phosphate buffer 0.1 M; 10% normal goat serum; 0.25% TX-100) 1 h. Endogenous biotins were blocked with the avidin & biotin blocking kit (Vector Labs, SP-2001) according to the manufacturer’s protocol. Sections were then incubated with anti-sacsin (Abcam; ab181190) or anti-neurofilament-H (NFH) antibody (Millipore, MAB5266) diluted in phosphate buffer 0.1 M; 1% normal goat serum; 0.25% TX-100 overnight at 4 °C. Sections were then incubated with appropriate biotinylated secondary antibodies (Vectors Labs) followed by VECTASTAIN ABC reagent for 1 h, washed, and reacted with VECTOR DAB substrate. Sections were dehydrated in a graded series of ethanol dilutions, cleared in xylene, counterstained with cresyl-violet or not and coverslipped using Protocol mounting medium (Fisher Scientific). Immunolabeling was performed simultaneously in at least three aged-matched animal per group. Mean grey values were collected using ImageJ in three different fixed-sized regions of interest per mouse for Purkinje cell dendrites and axons, as well as for the corpus callosum. For Purkinje cell bodies, mean grey values were collected from 8 to 10 cell bodies with a fixed-sized region of interest.

Immunolabeling of human-derived fibroblasts was performed as followed. Cells plated onto 12 mm round glass coverslips were fixed in ice-cold methanol 7 min at -20 °C. Cells were then washed with phosphate buffered-saline (PBS) three times. Cells were incubated 30 min in PBS; 5% normal goat serum. Cells were then incubated in the presence of primary monoclonal anti-vimentin antibody (1/4000, clone V9, SIGMA-Aldrich) diluted in PBS; 1% normal goat serum for 2 h at room temperature. Secondary anti-mouse Alexa-Fluor 555 antibody (ThermoFisher Scientific) was applied for 45 min.

Imaging was performed using Zeiss Axiovert M2 microscope or an Olympus IX81 inverted microscope with appropriate lasers using an Andor/Yokogawa spinning disk system (CSU-X), with a sCMOS camera using a 20×, 60× or a 100× objective lenses (NA1.4).

### Preparation of cerebellar tissue lysates and western blotting

We used our published protocols for the preparation of cerebellar protein extracts and western blot analysis [43]. Immunoblots were probed with polyclonal anti-sacsin (Abcam, ab181207, 1:2000) and monoclonal anti-vinculin (SIGMA-Aldrich, V9131, 1:1000).

### Behavioral test

Mice were tested for motor balance, motor coordination and muscle strength using the balance beam, rotarod and inverted grid tests. Female and male cohorts were tested at 45, 90 and 180 days of age (females *n* ≥ 7 per groups, males *n* ≥ 6 per groups) (females *n* ≥ 9 per groups, males n ≥ 7 per groups). Behavioural testing was performed as previously described in Lariviere et al. [22].

### Purkinje cell counts

Purkinje cell counts were performed as previously reported [43].

### Acute slice preparation

Animals between the ages of P90–100 were deeply anaesthetized with isofluorane, rapidly sacrificed, and brains were removed into ice-cold low-Ca^2+^ artificial cerebrospinal fluid (ACSF) that was bubbled with an O_2_/CO_2_ (95% / 5%) mixture as previously described [23, 44]. Sagittal cerebellar vermis slices (250 μm) were cut using a VT1200S microtome (Leica Microsystems, Germany). Slicing ACSF contained (in mM): NaCl, 125; KCl, 2.5; MgCl_2_, 4; NaH_2_PO_4_, 1.25; KCl, 2.5; MgCl_2_, 4; NaH_2_PO_4_, 1.25; NaHCO_3_, 26; CaCl_2_, 2; dextrose, 25; with a final osmolality of ~ 320 mOsm and pH 7.4. Slices were then transferred to ACSF that contained 1 mM MgCl_2_ and 2 mM CaCl_2_ (incubation and recording ACSF), were incubated at 37 °C and then cooled to room temperature where they were incubated in bubbled ACSF for up to an additional 6 h.

### Electrophysiology

Loose-cell attached recordings were made with a glass electrode pulled with a P-1000 puller (Sutter Instruments, Novato, CA, USA) filled with ACSF to record action potentials without dialyzing the intracellular solution and thereby altering intrinsic firing rates. Data was collected and analyzed off-line using custom acquisition and analysis routines with Igor Pro software (Wavemetrics, Portland, OR, USA). Statistical comparisons were made using Igor Pro or JMP (SAS, Cary, NC, USA) software. Data are represented as mean ± standard error of the mean (SEM), N = animal number, n = cell number.

### Statistical analysis

Data for the behavioral phenotyping are shown as the mean ± standard error of the mean (SEM). Beam and rotarod analyses were done using GraphPad Prism7 software and significance level was set at 0.05. Two-way ANOVA with repeated-measures was performed to assess the effect of time and genotype followed by Tukey’s post hoc pairwise comparisons. For all other statistical analyses, comparisons were made using unpaired Student *t*-test with significance level of 0.05.

## Additional file

Additional file 1: **Figure S1.** Generation of *Sacs*^R272C^ mice. (A) Targeting vector was constructed by first cloning the gene segment which includes exons 6 through 8 into PelleR B00001F7_G01 Ozgene proprietary plasmid containing a PGK-neo cassette flanked by two FRT sites, followed by site-directed mutagenesis for introduction of the R272C mutation in exon 7. Targeting vector was completed by incorporation of 6.3Kb 5′ and 3′ homology arms. Localization of forward and reverse primers for genotyping are identified on the knock-in allele. (B) PCR analysis of tail genomic DNA extracted from *Sacs*^R272C^, heterozygous (Het) and control (WT) mice. PCR amplicons migrate to 220 bp for the wild-type allele, whereas the R272C allele migrates to 330 bp. (TIF 4742 kb)

## Abbreviations

AIS: Axon initial segment
ARSACS: Autosomal recessive spastic ataxia of Charlevoix-Saguenay
CV: Coefficient of variation
DNA: Deoxyribonucleic acid
NF: Neurofilament
NFH: Neurofilament-heavy
PCR: Polymerase chain reaction
qRT-PCR: Quantitative reverse transcription polymerase chain reaction
RNA: Ribonucleic acid
*Sacs*^*−/−*^: *Sacs* knock-out mice
*Sacs*^R272C^: Mice homozygous for sacsin R272C mutation
