# Supplementary figures and images for: Sacs R272C missense homozygous mice develop an ataxia phenotype
Source: Mol Brain. 2019 Mar 12;12:19. doi: 10.1186/s13041-019-0438-3 (PMC6416858; doi:10.1186/s13041-019-0438-3)

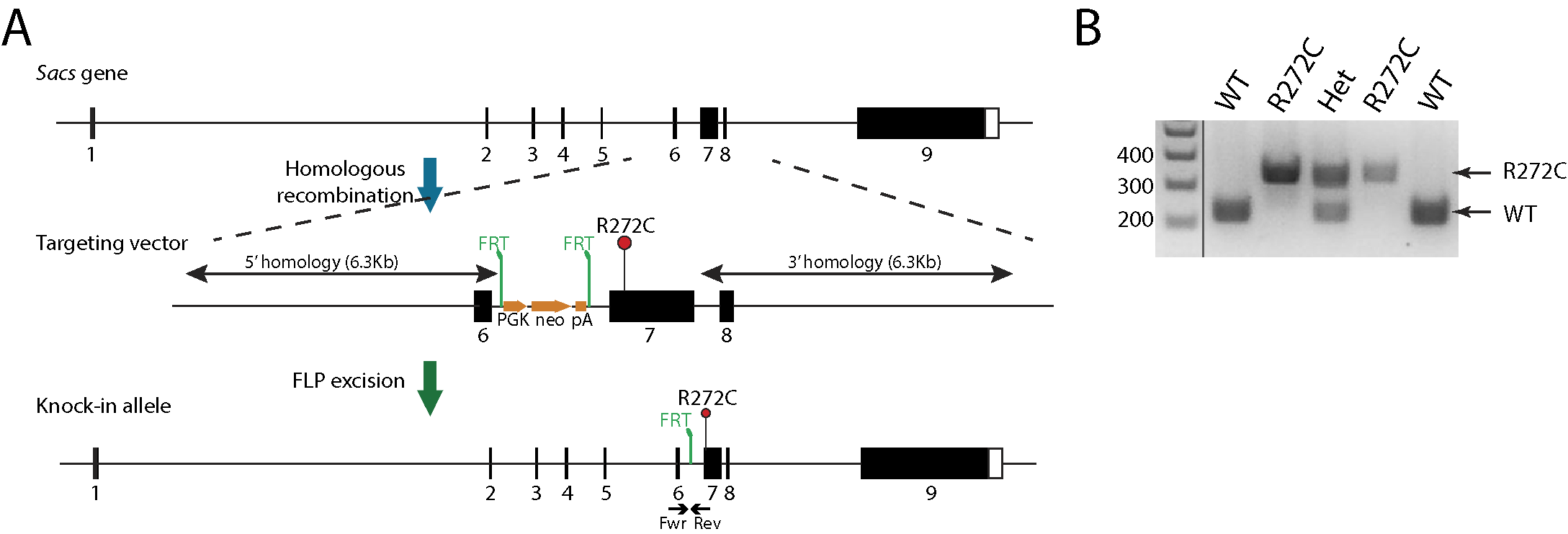

Supplement: Supplementary file 1 — Figure S1. Generation of SacsR272C mice. (A) Targeting vector was constructed by first cloning the gene segment which includes exons 6 through 8 into PelleR B00001F7_G01 Ozgene proprietary plasmid containing a PGK-neo cassette flanked by two FRT sites, followed by site-directed mutagenesis for introduction of the R272C mutation in exon 7. Targeting vector was completed by incorporation of 6.3Kb 5′ and 3′ homology arms. Localization of forward and reverse primers for genotyping are identified on the knock-in allele. (B) PCR analysis of tail genomic DNA extracted from SacsR272C, heterozygous (Het) and control (WT) mice. PCR amplicons migrate to 220 bp for the wild-type allele, whereas the R272C allele migrates to 330 bp. (TIF 4742 kb) [file 13041_2019_438_MOESM1_ESM.tif]
